# Diverse Roles of Cohesin in Chromosome Dynamics and Stem Cells

**DOI:** 10.3390/biotech15030070

**Published:** 2026-08-19

**Authors:** Eui-Hwan Choi

**Affiliations:** Department of Biotechnology, Korea National University of Transportation, Jeungpyeong 27909, Republic of Korea; ehchoi@ut.ac.kr

**Keywords:** cohesin, condensin, cell cycle, embryonic stem cells, chromosome organization, DNA repair, stem cell differentiation, rec8, cohesinopathies

## Abstract

The cohesin complex is a highly conserved, ring-shaped protein assembly that plays fundamental roles in chromosome biology. Originally identified as the molecular glue that holds sister chromatids together from DNA replication until cell division, cohesin has since been recognized as a pleiotropic regulator of genome organization, gene expression, DNA repair, and cell fate determination. In embryonic stem cells (ESCs), cohesin’s functions extend beyond canonical sister chromatid cohesion to include the maintenance of three-dimensional (3D) chromatin architecture through DNA loop extrusion, regulation of pluripotency-associated transcriptional programs, and facilitation of homologous recombination-mediated DNA repair during the prolonged S phase. Recent discoveries have revealed that meiosis-specific cohesin components, particularly the α-kleisin subunit REC8 and its interacting partner STAG3, are expressed and functionally active in mitotic ESC chromosomes, where they contribute to chromosomal organization and sister chromatid cohesion in concert with mitotic RAD21-containing cohesin. Furthermore, the interplay between cohesin and condensin complexes at shared genomic binding sites has emerged as a critical determinant of chromosome topology, with cohesin depletion leading to aberrant condensin accumulation and chromosome hypercompaction. Importantly, perturbations in cohesin function not only impair ESC self-renewal but also direct lineage-specific differentiation, linking cohesin to stem cell fate determination. Germline mutations in cohesin and its regulators underlie a spectrum of developmental disorders termed cohesinopathies, while somatic mutations are frequently observed in various cancers. This review provides a comprehensive overview of the diverse roles of cohesin in chromosome structure, cell cycle regulation, and stem cell biology, with particular emphasis on recent findings in ESCs that illuminate the complex interplay between mitotic and meiotic cohesin complexes.

## 1. Introduction the Cohesin Complex and Its Diverse Roles in Genome Integrity

The faithful transmission of genetic information from one cell generation to the next is a fundamental requirement for all living organisms. Central to this process is the cohesin complex, a ring-shaped protein assembly that was originally identified for its essential role in holding sister chromatids together following DNA replication until their separation at anaphase [1,2]. Since its initial characterization, cohesin has emerged as a multifunctional molecular machine that participates in a remarkably diverse array of cellular processes, including chromosome condensation, DNA damage repair, transcriptional regulation, and three-dimensional (3D) genome organization [3,4,5].

**Figure 1:**
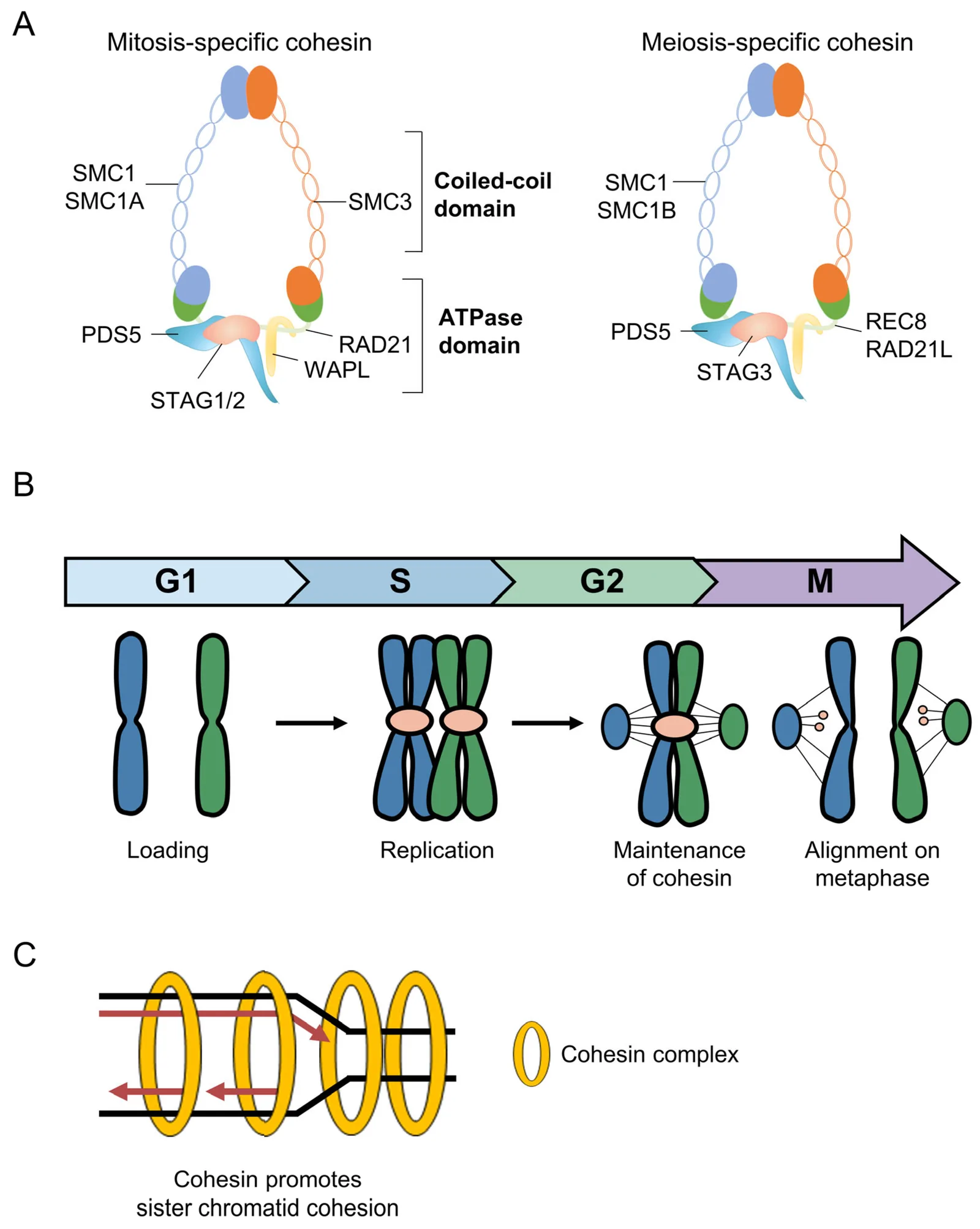
Cohesin architecture and canonical sister chromatid cohesion. (**A**) Schematic comparison of mitosis-specific and meiosis-specific cohesin complexes, showing the substitution of core subunits and associated regulatory proteins in the two assemblies. (**B**) Cell-cycle-dependent cohesin dynamics, illustrating loading in G1, cohesion establishment during DNA replication in S phase, maintenance through G2, and chromosome alignment at metaphase. (**C**) Ring-shaped cohesin encircles sister chromatids to promote sister chromatid cohesion.

The core cohesin complex in vertebrates consists of four subunits: two structural maintenance of chromosomes (SMC) proteins, SMC1A and SMC3, which form long coiled-coil arms joined at a hinge domain; the α-kleisin subunit RAD21, which bridges the ATPase head domains of SMC1A and SMC3 to close the ring; and a stromal antigen (STAG) protein, either STAG1 or STAG2, which associates with RAD21 (Figure 1A) [1,6]. Additional regulatory proteins, including NIPBL-MAU2 (the cohesin loading complex), WAPL (the cohesin release factor), PDS5A/B, and Sororin, dynamically modulate cohesin’s association with chromatin throughout the cell cycle [7,8,9]. In meiotic cells, specialized cohesin subunits replace their mitotic counterparts: SMC1β substitutes for SMC1A, REC8 or RAD21L replaces RAD21 as the α-kleisin, and STAG3 replaces STAG1/STAG2 (Figure 1A) [10,11]. These meiosis-specific components confer unique properties required for homologous chromosome pairing, synapsis, and meiotic recombination [12,13]. Embryonic stem cells (ESCs) represent a unique cellular context in which to study cohesin function. Derived from the inner cell mass of blastocyst-stage embryos, ESCs possess two defining characteristics: unlimited self-renewal capacity and pluripotency, the ability to differentiate into all cell types of the embryo proper [14,15]. ESCs exhibit distinctive cell cycle features, including a truncated G1 phase and a prolonged S phase, which are intimately linked to the maintenance of their pluripotent state (Figure 1B) [16,17]. The chromatin landscape of ESCs is characterized by a globally open configuration that supports the expression of diverse gene programs, including, remarkably, meiosis-specific cohesin components [18,19]. Recent studies have demonstrated that these meiotic cohesins are not merely passively expressed but are functionally active in mitotic ESC chromosomes, adding new dimensions to our understanding of cohesin biology [18].

This review provides a comprehensive examination of the roles of cohesin in chromosome organization, cell cycle regulation, DNA repair, and stem cell fate determination. We discuss recent advances in understanding the mechanisms of cohesin-mediated DNA loop extrusion and its implications for transcriptional regulation. We highlight the discovery of functional meiotic cohesin in mitotic ESCs and explore the interplay between cohesin and condensin in chromosome topology. Finally, we consider the implications of cohesin dysfunction in developmental disorders and cancer, providing a holistic view of this essential chromosomal complex.

## 2. Cohesin-Mediated Chromosome Structure and Topological Organization

### 2.1. DNA Loop Extrusion—Mechanisms and Regulation

One of the most significant conceptual advances in chromosome biology in recent years has been the recognition that cohesin functions as a molecular motor capable of extruding DNA loops [20,21]. This loop extrusion activity is central to the establishment of topologically associating domains (TADs), which are megabase-scale regions of preferential chromatin interactions that organize the genome into functional compartments [22,23]. Single-molecule experiments have demonstrated that cohesin, in complex with its loader NIPBL, can processively extrude DNA at rates of up to 1 kb per second, generating progressively larger loops until it encounters a barrier [20,24]. The primary barrier to cohesin-mediated loop extrusion is the CCCTC-binding factor (CTCF), an 11-zinc finger protein that binds to specific DNA sequences throughout the genome [25,26]. The orientation of CTCF binding is critical: cohesin is stalled when it encounters the N-terminal face of a CTCF molecule but continues or even accelerates extrusion when approaching from the C-terminal side [27]. This directional sensitivity underlies the convergence rule observed at the majority of DNA loops, where anchor sites are occupied by CTCF molecules in convergent orientations [28]. The interaction between cohesin and CTCF is mediated primarily through the SMC1A hinge domain and the STAG subunit, with the STAG protein specifically engaging an interface on CTCF to arrest loop extrusion [29,30].

Recent studies have identified additional factors that regulate DNA loop extrusion dynamics. The tension applied to the DNA fiber modulates the efficiency of CTCF as a barrier, high tension enhances CTCF blocking ability, while low tension permits cohesin to bypass even properly oriented CTCF sites [27]. RNA-DNA hybrids (R-loops), which form during transcription, can also impede loop extrusion in vitro and correlate with cohesin localization in vivo [31,32]. Furthermore, G-quadruplex structures that form at R-loops can enhance CTCF binding to proximal motifs, thereby increasing DNA loop formation [33]. Transcription-induced DNA supercoiling represents yet another regulator, with torsional strain capable of accelerating the rate of cohesin extrusion [34]. MCM complexes, which license eukaryotic origins of DNA replication, have also been shown to impede cohesin-mediated loop extrusion during interphase through specific protein–protein interactions [35]. These findings collectively indicate that the dynamics of DNA loop extrusion are governed by a complex interplay of protein barriers, nucleic acid structures, and the biophysical properties of the chromatin fiber.

### 2.2. Cohesin at Cis-Regulatory Elements and Transcriptional Control

The localization of cohesin to cis-regulatory elements, enhancers, promoters, and insulator sites profoundly influences gene transcription. A cohesin traffic pattern has been described in which cohesin loads at transcriptionally active enhancers, translocates along chromatin until stalling at CTCF sites or transcription start sites, and is subsequently removed from DNA at transcription termination sites [36]. This dynamic cycle of loading, translocation, and removal ensures the proper spatial communication between enhancers and their target promoters. Cohesin directly regulates transcription through multiple mechanisms. It can increase RNA polymerase II (Pol II) occupancy at genes and promote the transition of paused Pol II to the actively elongating state [37]. CTCF, working in conjunction with cohesin, directs enhancer–promoter communication and can suppress antisense transcription at divergently oriented promoters [38,39]. Loss of promoter-proximal CTCF binding alters transcription of cell type-specific genes and disrupts long-range enhancer–promoter interactions [40]. Additionally, the mediator complex plays a role in maintaining cohesin at enhancers; loss of mediator induces cohesin relocalization from enhancers to CTCF sites, thereby reducing enhancer–promoter interactions and gene expression [41]. Intriguingly, studies employing acute degradation of cohesin or CTCF have revealed that DNA loops and domains decrease rapidly upon protein loss, yet nascent transcription is only minimally perturbed in the short term [42,43]. Higher-resolution assays such as Micro-C have shown that some enhancer–promoter interactions can persist in the absence of cohesin or CTCF, suggesting the existence of loop extrusion-independent mechanisms of enhancer–promoter communication [44,45]. These observations have prompted a re-evaluation of the precise role of cohesin-mediated loops in transcriptional regulation, with current models suggesting that cohesin may function to reinforce or stabilize pre-existing enhancer–promoter contacts rather than being absolutely required for their formation.

Quantitative measurements have further refined our understanding of DNA loop dynamics. Super-resolution live-cell imaging and single-molecule assays indicate that cohesin-mediated loops are relatively rare in the population, existing in the looped state only approximately 5% of the time, with half-lives of 10–30 min [46,47]. The transient nature of these loops raises important questions about how such short-lived structures can exert lasting effects on gene expression, a paradox that remains an active area of investigation.

### 2.3. Nucleosome Organization and Chromatin Assembly in Cohesin-Mediated Loop Formation

Loop extrusion and cis-regulatory control operate on a chromatin template packaged into nucleosomes. Consequently, nucleosome positioning and turnover regulate cohesin loading, translocation distance, and loop formation efficiency. Single-molecule imaging confirms that nucleosomes act as physical obstacles to cohesin translocation [48]. Reconstituted human cohesin-NIPBL compacts both naked and nucleosome-bound DNA via loop extrusion, though nucleosomes reduce reaction efficiency [49]. Consistently, removing nucleosomes markedly enhances cohesin- and condensin-mediated loop extrusion in Xenopus egg extracts [50]. Cohesin loading requires nucleosome-free regions. Specifically, the Remodels the Structure of Chromatin (RSC) remodeling complex generates nucleosome-depleted DNA, which directly enables NIPBL-dependent cohesin loading and links chromatin accessibility to genome recruitment [51]. These findings position nucleosome positioning and remodeling as upstream determinants of loop-extrusion dynamics.

During transcription, the interplay between nucleosomes and cohesin is particularly dynamic. Elongating RNA polymerase II continuously disassembles and reassembles nucleosomes in its wake, and this transcription-coupled remodeling repositions cohesin, pushing translocating complexes toward transcription termination sites or convergent CTCF boundaries [36,37]. Cohesin loads at nucleosome-free promoters and active enhancers. Consequently, transcription-defined nucleosome organization dictates cohesin entry sites and biases its translocation direction, coupling transcription to local loop architecture.

DNA replication transiently displaces parental nucleosomes during replisome progression and requires their rapid redeposition behind the replication fork. Replication-coupled chromatin assembly, mediated principally by the histone chaperone chromatin assembly factor 1 (CAF-1), restores the nucleosome template on nascent DNA and is intimately coupled to the establishment of sister-chromatid cohesion [52]. During S phase, the replisome-associated acetyltransferase ESCO2 acetylates the SMC3 subunit of cohesin [35]. This acetylation stabilizes cohesin at the replication fork, converting it into a replication-competent state [53]. Post-replicative nucleosome deposition and acetylation-dependent cohesin stabilization occur simultaneously, ensuring newly replicated sister chromatids are packaged into chromatin and topologically linked. This coupling is especially relevant to the prolonged S phase of ESCs, in which the extended replication program places heavy demands on both chromatin assembly and cohesion establishment.

Under conditions of replication stress, the relationship between nucleosome dynamics and cohesin acquires additional importance. Cohesin is actively recruited to stalled replication sites in a manner dependent on the Rad50/MRN complex, where it stabilizes the stalled fork and promotes its restart [54]. Stalled forks can be remodeled into four-way reversed-fork structures whose formation and protection depend on RAD51 and associated fork-remodeling factors [55]; the resulting regressed arm must be re-packaged into nucleosomes and serves as a substrate for homologous recombination (HR)-mediated restart. Cohesin contributes to this process by holding the nascent sister chromatid in register with its template, thereby facilitating the homology-directed events that underlie fork restart, whereas the controlled, WAPL-dependent removal of cohesin is subsequently required to resume DNA synthesis at restarted forks [56]. Because ESCs rely on HR to resolve replication stress during S phase, coupling chromatin assembly, cohesin loading, and fork-associated recombination is critical for genome stability in pluripotency. Nucleosome organization and chromatin assembly actively regulate cohesin-dependent looping during transcription and replication, and this regulation is dynamically reshaped under replication stress.

## 3. Cohesin and Cell Cycle Regulation in Embryonic Stem Cells

### 3.1. Unique Cell Cycle Features of ESCs

ESCs exhibit a distinctive cell cycle architecture that differs markedly from that of differentiated somatic cells [16,57]. Mouse ESCs (mESCs) undergo rapid cell division with a generation time of approximately 12 h, roughly half that of typical somatic cells [58]. The most striking feature of the ESC cell cycle is the extremely short G1 phase, lasting only about 3 h, with approximately 65% of asynchronous cells residing in S phase compared to only 15% in G1 (Figure 1B) [58,59]. Human ESCs display a similar trend, though with a somewhat longer generation time of 15–16 h and a slightly lower proportion of cells in S phase (~50%) [17,60]. The molecular basis for the abbreviated G1 phase in ESCs involves constitutive expression of cyclins E, A, and B and sustained activity of CDK1 and CDK2 throughout the cell cycle [61,62]. Unlike somatic cells, ESCs lack significant expression of cyclin D, which in differentiated cells mediates the response to mitogenic signals during G1 [58]. The continuous activity of cyclin E-CDK2 maintains retinoblastoma protein (Rb) in a hyperphosphorylated and therefore inactive state throughout the ESC cell cycle, effectively eliminating the Rb-dependent restriction point that normally governs the G1-to-S phase transition [57,62]. This truncated G1 phase is believed to protect pluripotency by minimizing the window during which differentiation-inducing signals can act [63].

### 3.2. Cohesin, DNA Repair, and Genome Integrity in ESCs

The prolonged S phase of ESCs creates both opportunities and challenges for genome maintenance. The extended time spent in S phase allows for the utilization of (HR), a high-fidelity DNA repair pathway that operates predominantly during S and G2 phases when sister chromatids are available as repair templates (Figure 1B,C) [64,65]. On the other hand, the rapid transit through G1 means that ESCs may enter S phase before completing DNA repair or replication licensing, generating constitutive replication stress [66]. ESCs address these challenges through the elevated expression of HR-related proteins, including RAD51, RAD52, and RAD54, throughout the entire cell cycle [64,67]. These factors are approximately six-fold more abundant in ESCs than in somatic cells, enabling rapid localization to DNA break sites and efficient repair of double-strand breaks (DSBs) and collapsed replication forks [64,68]. The cohesin complex plays a critical supporting role in this process. Sister chromatid cohesion, maintained by cohesin from S phase through mitosis, is essential for HR-mediated DNA repair because it physically constrains the sister chromatid in proximity to the damaged chromosome, facilitating the homology search step of recombination [69,70].

Knockdown of cohesin components in ESCs has been shown to cause significant defects in DNA replication and repair. Depletion of either RAD21 or REC8 leads to higher rates of premature sister chromatid separation, delayed replication fork progression, and increased susceptibility to DNA damage [18]. These replication defects can trigger G2/M arrest and promote apoptosis, underscoring the essential role of cohesin in maintaining the rapid proliferation characteristic of ESCs [18,71]. Furthermore, DNA fiber assays have demonstrated that cohesin-depleted ESCs exhibit reduced fork speed and increased fork stalling, consistent with a role for cohesin in stabilizing replication forks and preventing their collapse [18]. The relationship between cohesin and genome integrity has additional implications for ESC biology. During differentiation, the expression levels of HR factors decrease progressively, and ESCs switch their preferred DNA repair pathway from HR to non-homologous end joining (NHEJ) [64,72]. This switch is accompanied by changes in cohesin expression and localization, suggesting that the coordinated regulation of cohesin and HR factors is integral to the transition from the pluripotent to the differentiated state [58]. Interestingly, ectopic expression of HR factors can enhance the efficiency of ESC differentiation by promoting the repair of DNA breaks that arise during chromatin remodeling, highlighting the intimate connection between genome integrity maintenance and cell fate transitions [64].

## 4. Meiosis-Specific Cohesin Components REC8 and STAG3 in Mitotic ESC Chromosomes

An interesting recent development in stem cell biology is the unexpected identification of meiosis-specific cohesin components, particularly REC8 and STAG3, in mitotically proliferating ESCs. Unlike the canonical functions of meiotic cohesins, which have been extensively characterized during meiosis, their proposed mitotic roles in pluripotent stem cells have only recently emerged and remain at an early stage of investigation. Consequently, current evidence remains limited and relies primarily on a small number of studies in mouse ESCs, including several from a few research groups. We therefore acknowledge that this section necessarily relies on a restricted evidence base and that many mechanistic conclusions have not yet been independently validated by search groups.

This limited evidence does not necessarily reflect selective citation but rather the current developmental stage of the field. Mitotic functions of REC8/STAG3 in ESCs represent a recently identified biological phenomenon, and relatively few laboratories have investigated whether meiosis-specific cohesin complexes acquire functional roles outside meiosis. At present, independent confirmation in human embryonic stem cells, induced pluripotent stem cells, or additional mammalian model systems remains unavailable. Therefore, the concepts discussed below should be interpreted as an emerging model that is supported by current experimental evidence but still awaits broader validation across different biological systems.

Nevertheless, several independent observations provide indirect biological plausibility for this hypothesis. Pluripotent stem cells are known to exhibit an unusually open chromatin landscape, permissive transcriptional activity, and transient expression of numerous germline- and meiosis-associated genes that are normally silenced in differentiated somatic cells [73,74,75]. These epigenetic features distinguish ESCs from other cell types and provide a mechanistic framework for the expression of REC8 and STAG3. Although such observations do not directly demonstrate mitotic functions of meiotic cohesin, they support the possibility that pluripotent cells exploit components of the meiotic program to satisfy their exceptional requirements for genome stability, HR, and chromosomal organization during rapid self-renewal.

Accordingly, the following sections summarize the currently available evidence while explicitly distinguishing experimentally demonstrated findings from mechanistic interpretations and future hypotheses. Additional studies using endogenous protein tagging, genome-wide chromatin occupancy analyses, and human pluripotent stem cell models will be essential for determining the generality and physiological significance of this proposed mitotic cohesin program.

### 4.1. Expression and Localization of Meiotic Cohesin in ESCs

One of the most unexpected discoveries in recent ESC biology has been the finding that meiosis-specific cohesin components are expressed at substantial levels in mitotic ESCs [18,71]. While differentiated somatic cells such as mouse embryonic fibroblasts (MEFs) express only mitotic cohesin subunits, ESCs express significant levels of the meiotic α-kleisin REC8 and the meiosis-specific STAG protein STAG3 [18]. Both immunoblotting and RNA sequencing analyses have confirmed that REC8 and STAG3 are abundantly expressed in mESCs at levels comparable to their mitotic counterparts [18,71]. Importantly, the expression of other meiosis-specific cohesin components, such as SMC1β and RAD21L, is minimal or absent in ESCs, indicating selective rather than global activation of the meiotic cohesin program [18].

The globally open chromatin configuration of ESCs, characterized by low levels of heterochromatin marks and high transcriptional activity across broad genomic regions, likely underlies the expression of these typically meiosis-restricted genes [76,77]. This permissive chromatin state allows ESCs to express a diverse repertoire of genes that may confer functional advantages during rapid proliferation and genome maintenance.

Pluripotent cells express genes normally restricted to meiosis, as reported in other studies. A study of pre-meiotic marker genes across mouse pluripotent cell types showed comparable expression in ESCs, iPSCs, and pluripotent cells, driven by low-level transcription in open chromatin [74]. This independent observation is consistent with, but does not by itself establish, a specific structural or functional role for REC8 and STAG3 of the kind proposed in this section; we return to this distinction, and its implications for interpreting the ESC phenotypes described below, in the Discussion.

### 4.2. REC8-STAG3 Interaction and Nuclear Import

In meiotic cells, STAG3 is known to be essential for the stabilization and chromosomal localization of REC8 [78,79]. A similar relationship exists in ESCs: STAG3 directly interacts with REC8 and is required for the efficient translocation of REC8 from the cytoplasm to the nucleus [18]. Immunoprecipitation experiments have demonstrated that REC8 and STAG3 form a physical complex in ESCs, and knockdown of STAG3 results in a marked reduction in nuclear REC8 levels [18]. Conversely, overexpression of STAG3 enhances REC8 nuclear accumulation, confirming that STAG3 acts as a stabilizing factor and nuclear import facilitator for REC8 in the mitotic context [18]. This mechanism mirrors the STAG3-dependent stabilization of REC8 observed during meiotic prophase, suggesting conservation of this regulatory relationship across distinct cellular contexts.

### 4.3. Distinct and Overlapping Roles of RAD21 and REC8 Cohesins

A key mechanistic question arising from the co-expression of mitotic and meiotic cohesins in ESCs is whether they function as a single system with interchangeable subunits or as genuinely parallel pathways. Three lines of independent evidence argue for the latter.

First, immunoprecipation analyses confirm that they form separate complexes with the core subunit SMC3 (SMC3-RAD21 and SMC3-REC8) and never co-assemble into a single ring [18]. Second, high-resolution 3D-structured illumination microscopy (3D-SIM) shows that they exhibit distinct loading patterns and load at largely non-overlapping positions along the chromosomal axis, a spatial segregation that becomes especially clear upon WAPL depletion [18]. Third, and most informative mechanistically, co-depletion of RAD21 and REC8 produces an additive, not epistatic, increase in premature sister-chromatid separation [18,71]. Additivity is the quantitative signature of two parallel pathways acting on the same phenotype, whereas an epistatic interaction would indicate a single shared pathway. While both populations contribute to cohesion, these lines of evidence demonstrate that the two types of cohesin act cooperatively rather than redundantly.

We interpret these data through the biophysics of meiotic cohesin. In meiosis, REC8-cohesin is the more stable, protease-protected kleisin. It is selectively shielded from separase at centromeres by the shugoshin-PP2A system and exhibits markedly slower turnover than mitotic RAD21-cohesin, which is highly dynamic and acutely WAPL-sensitive, with chromatin residence times on the order of minutes [80]. Transposing these intrinsic properties into the ESC nucleus yields a concrete division. REC8-cohesin is well suited to provide a stable, long-residence structural cohesion pool, precisely what is required to hold a sister chromatid in register throughout the unusually long ESC S-phase and to maintain a damaged locus tethered to its template during an extended homology search in HR [80]. RAD21-cohesin, by contrast, is well suited to provide a fast-cycling regulatory pool that supports rapid loop turnover, enhancer–promoter reorganization, and the continuous topological remodelling demanded by the hyperdynamic pluripotent genome [72]. The two kleisins thus partition a single problem that no single kleisin could solve, because stability and dynamism are mutually exclusive properties of one molecule.

This framework also refines the compensatory plasticity of the system. Previous studies noted that depletion of RAD21 leads to an increase in the proportion of SMC3-REC8 complexes, suggesting a compensatory mechanism [18]. Specifically, immunofluorescence analyses confirmed that RAD21 knockdown results in an approximately 1.9-fold increase in nuclear REC8 levels and a corresponding increase in REC8-SMC3 interaction [71]. In our reading, this is not passive compensation but direct evidence that the two pools are coupled through two shared, limiting resources: a common SMC3 core and a STAG3-gated nuclear-import step. When RAD21 is lost, SMC3 is captured by REC8, and the resulting REC8 complex accumulates in the nucleus only to the extent that STAG3 permits its import; because the increase is observed predominantly in the nuclear fraction, it most parsimoniously reflects STAG3-facilitated import and post-translational stabilization rather than transcriptional induction. The system therefore behaves as a partially buffered, mutually constraining pair, and its set-point is determined by the relative abundances of SMC3, the two kleisins, and STAG3.

Why house both complexes in one mitotic cell at all? We propose that coexistence is an adaptive solution to a biophysical trade-off that is especially acute in ESCs. A pluripotent cell must protect its genome integrity during rapid proliferation, which requires stable cohesin. It must also maintain a flexible 3D genome to allow transcription and cell fate transitions, which requires dynamic cohesin [66,67]. These demands cannot be optimized by a single kleisin with a single chromatin association duration, but they can be met by two parallel-operating kleisins with different chromatin binding stabilities.

This repositions the unexpected expression of meiotic cohesin in mitotic ESCs not as a curiosity but as a plausible mechanism for decoupling genome protection from dynamic topological regulation [8]. The model further generates measurable predictions; stabilizing RAD21 should impair the rapid topological reorganization that accompanies differentiation, whereas selective loss of REC8 should preferentially sensitize ESCs to replication stress and HR-dependent damage without equally affecting steady-state loop architecture (Table 1) [18,71,80].

## 5. Interplay Between Cohesin and Condensin in Chromosome Compaction

### 5.1. Shared Genomic Binding Sites of Cohesin and Condensin

Condensin is a related SMC complex that plays essential roles in chromosome compaction during mitosis [86,87]. Like cohesin, condensin forms a ring-shaped structure composed of SMC2 and SMC4 core subunits along with regulatory non-SMC subunits. Two types of condensin complexes exist in vertebrates—condensin I (containing CAP-D2, CAP-G, and CAP-H) and condensin II (containing CAP-D3, CAP-G2, and CAP-H2)—which have distinct but complementary roles in mitotic chromosome assembly [88,89]. Condensin mediates chromosome compaction through ATP-dependent positive supercoiling of DNA, which is thought to drive the initial steps of mitotic chromosome condensation [90]. A surprising finding from genome-wide binding analyses is that cohesin and condensin occupy highly overlapping genomic sites in ESCs [71,80,91]. ChIP-seq profiling has demonstrated that the mitotic cohesin components SMC3 and RAD21, the meiotic cohesin component REC8, and the condensin core factor SMC4 all bind to similar genomic locations that are enriched at CTCF binding sites [71]. Furthermore, these shared binding sites overlap with those of the pluripotency transcription factors OCT4 and NANOG, suggesting a potential link between chromosome topology and stem cell identity [71,80]. Co-immunoprecipitation experiments have confirmed that SMC3, RAD21, REC8, and SMC4 all physically interact with CTCF in ESCs, and these interactions are maintained throughout the cell cycle [71].

### 5.2. Cohesin Depletion Induces Condensin Accumulation and Chromosome Hypercompaction

**Figure 2:**
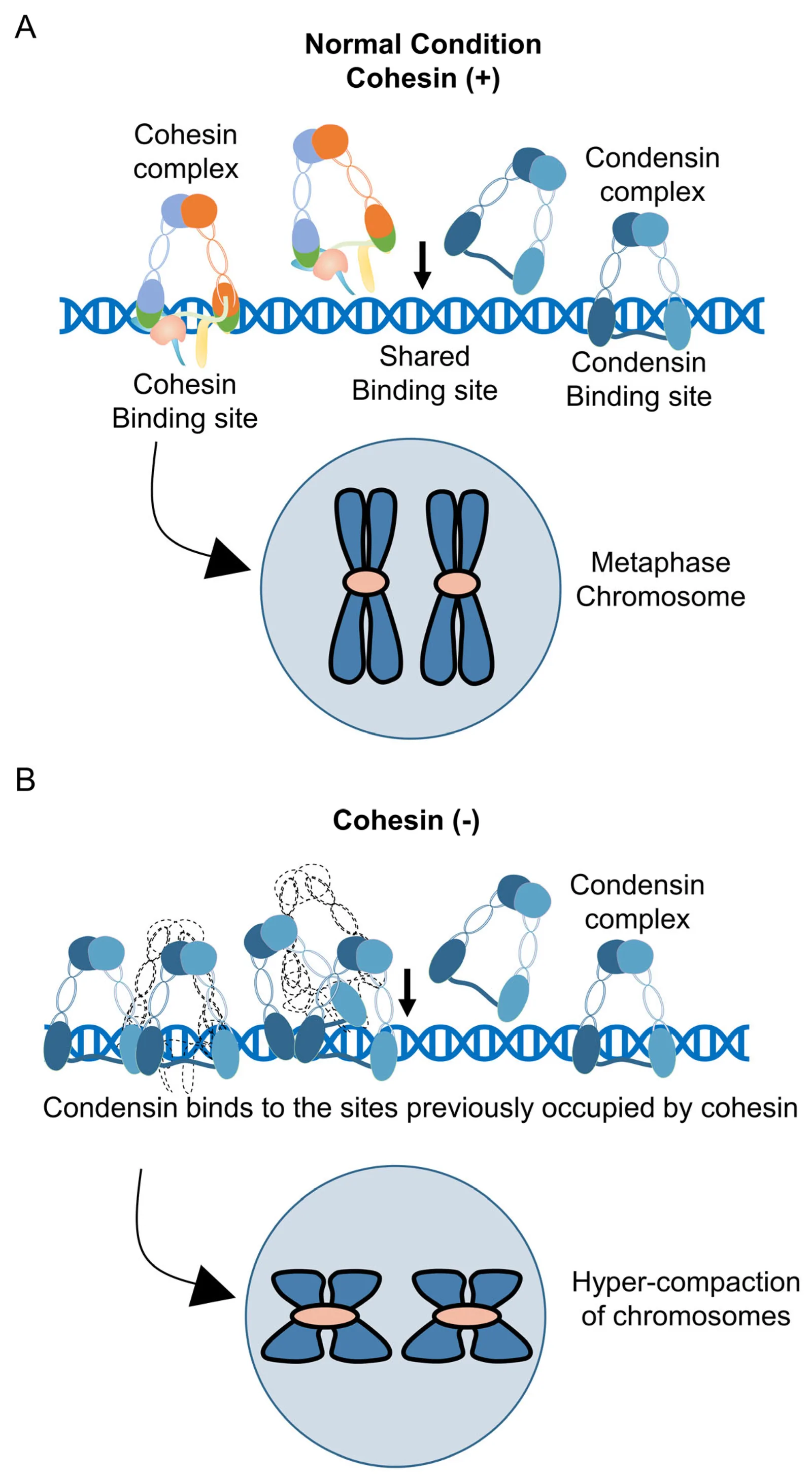
Interplay between cohesin and condensin in chromosome compaction. (**A**) Under normal conditions, cohesin and condensin occupy distinct and shared chromatin binding sites to support proper metaphase chromosome organization. (**B**) When cohesin is depleted, condensin redistributes to sites previously occupied by cohesin, resulting in excessive chromosome compaction and hypercondensation.

The functional relationship between cohesin and condensin becomes apparent when cohesin is depleted. Knockdown of the cohesin core subunit SMC3 in ESCs results in a significant reduction in SMC4 protein expression at the total cellular level [71]. However, a more nuanced picture emerges from subcellular fractionation experiments: while cytoplasmic SMC4 levels decrease upon SMC3 depletion, nuclear SMC4 levels paradoxically increase [71]. This nuclear accumulation of condensin is accompanied by the formation of punctate structures on compacted chromosomes, suggesting that condensin redistributes to cohesin-vacated binding sites on chromatin (Figure 2A) [71]. Time-course experiments following cell cycle synchronization have provided further insights into the dynamics of this redistribution. In control cells, nuclear SMC4 levels increase gradually following release from a double-thymidine block, reflecting the normal progression of condensin loading during S and G2 phases. In SMC3-depleted cells, however, nuclear SMC4 accumulation is markedly accelerated, reaching peak levels by 6 h post-release well before the normal condensin loading peak [71]. These findings suggest that cohesin normally restricts the premature loading of condensin onto chromosomes, and that the loss of this restraint leads to precocious and excessive chromosome compaction.

The hypercompaction phenotype observed upon cohesin depletion has been linked to the retinoblastoma protein (Rb)–condensin axis. Studies using high-resolution 3D-SIM imaging have demonstrated that knockdown of mitotic or meiotic cohesin components in ESCs leads to hyperloading of Rb–condensin II complexes onto chromosomes from prophase onward, resulting in the formation of shorter and thicker chromosomes [18]. Quantitative analyses have confirmed that the intensity and spatial density of SMC4 per chromosome area are significantly increased in cohesin-depleted ESCs compared to controls [18]. Notably, this hypercompaction phenotype is specific to ESCs and is not observed in MEFs, where meiotic cohesin components are not expressed (Figure 2A,B) [18]. This specificity suggests that the balance between mitotic and meiotic cohesins plays a unique role in regulating chromosome compaction in ESCs.

These observations are consistent with a model in which cohesin and condensin compete for overlapping binding sites on chromosomes. During normal cell cycle progression, the sequential loading and removal of cohesin during prophase creates binding sites for condensin, enabling the orderly transition from interphase chromosome organization to the highly compacted mitotic state [92,93]. When this transition is disrupted by premature cohesin loss, condensin can bind prematurely and excessively, leading to aberrant chromosome compaction that may compromise faithful chromosome segregation and gene expression.

## 6. Cohesin in Stem Cell Fate Determination and Implications for Cohesinopathies

### 6.1. Cohesin Maintains the Balance Between Self-Renewal and Differentiation

**Figure 3:**
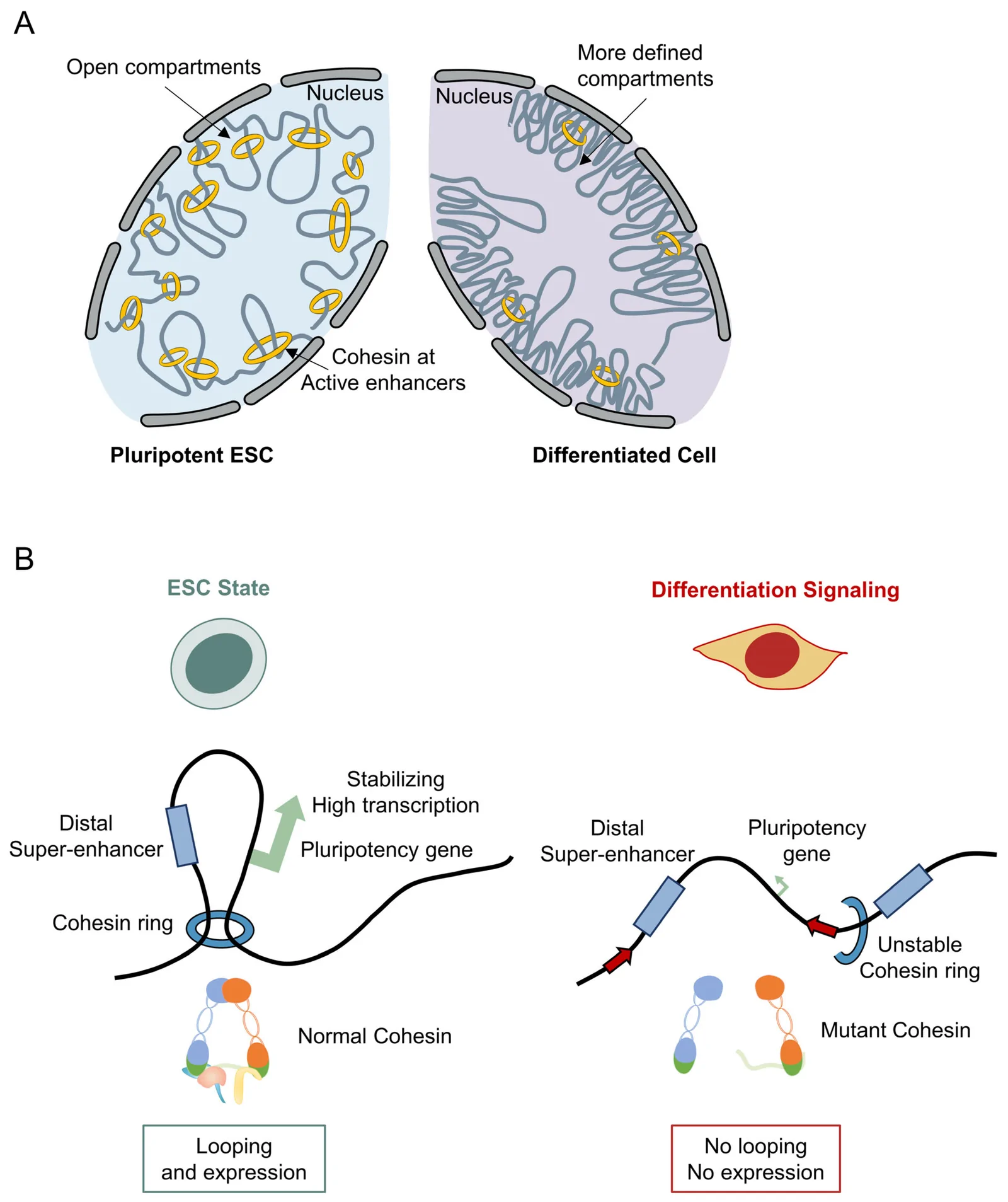
Cohesin-mediated chromatin organization and transcriptional regulation in embryonic stem cells. (**A**) In pluripotent embryonic stem cells (ESCs), cohesin is enriched at active enhancer regions within an open chromatin environment, whereas differentiated cells display more defined chromatin compartmentalization and reduced enhancer-associated cohesin activity. (**B**) Cohesin supports long-range communication between a distal super-enhancer and a pluripotency gene such as *Oct4* or *Nanog*, thereby stabilizing high transcription; upon differentiation signaling, cohesin occupancy is reduced and transcriptional output declines.

The connection between cohesin and stem cell pluripotency was first suggested by genome-wide binding studies showing that cohesin co-localizes with the mediator complex and pluripotency transcription factors at enhancers and core promoter regions of active genes in ESCs (Figure 3A) [80]. Subsequent functional studies have demonstrated that reduced cohesin expression impairs ESC self-renewal and promotes differentiation [19,94]. Knockdown of the cohesin subunits REC8, RAD21, or SMC3 in mESCs results in decreased expression of the key pluripotency markers OCT4, NANOG, SOX2, and KLF4, with particularly dramatic reductions observed upon RAD21 or SMC3 depletion (Figure 3B) [19]. Morphologically, cohesin-depleted ESCs display accelerated differentiation, transitioning from the characteristic dome-shaped colony morphology to a flattened, spread morphology approximately 12 h faster than control cells cultured without leukemia inhibitory factor (LIF) [19].

Transcriptomic analyses have provided deeper insights into the relationship between cohesin and ESC differentiation. RNA sequencing of cohesin-depleted ESCs has revealed hundreds of differentially expressed genes, with commonly upregulated genes enriched in biological processes related to embryonic development, cell division, and DNA methylation, while commonly downregulated genes are associated with chromatin organization, cell fate commitment, and translation [19]. These gene expression changes are consistent with a model in which cohesin normally functions to maintain the transcriptional programs that support ESC self-renewal, and its loss destabilizes pluripotency and promotes the activation of differentiation programs.

#### Mechanisms Underlying Cohesion Reduction During Differentiation

While cohesin depletion promotes ESC differentiation, how cohesin is reduced during differentiation remains less clear. Cohesin reduction results from integrated changes in chromatin accessibility, loading dynamics, and transcription during exit from pluripotency.

Stem cell differentiation involves remodeling of the pluripotent chromatin. ESCs feature accessible chromatin enriched with enhancers, super-enhancers, and promoter interactions that sustain *OCT4*, *SOX2*, and *NANOG* expression. As differentiation proceeds, these pluripotency-associated regulatory elements become progressively inactivated, while lineage-specific enhancers are established. Because cohesin is preferentially recruited to active enhancers through interactions involving the Mediator complex, reduced enhancer activity is expected to decrease cohesin occupancy at many pluripotency-associated loci [5]. Cohesin redistributes to drive the shift from pluripotency to lineage-specific gene expression, rather than undergo global loss.

Changes in cohesin dynamics are also regulated through alterations in its loading and release machinery. Chromatin loading of cohesin depends primarily on the NIPBL-MAU2 complex, whereas WAPL promotes cohesin release from chromatin by opening the cohesin ring. During differentiation, reduced cohesin loading together with increased cohesin turnover is thought to shorten cohesin residence time on chromatin, thereby facilitating extensive reorganization of chromatin loops and TADs. Experimental studies have shown that modulation of the balance between NIPBL-dependent loading and WAPL-mediated release alters chromatin architecture and transcriptional programs without necessarily changing total cohesin protein abundance, indicating that cohesin occupancy is dynamically regulated during developmental transitions [95].

Differentiation is additionally accompanied by widespread reorganization of 3D genome architecture. Super-enhancers are dismantled, chromatin interactions are reorganized, and enhancer–promoter contacts are reshaped in a lineage-specific manner. Because cohesin functions as the principal loop extrusion factor responsible for maintaining these chromatin interactions, the extensive remodeling of chromosomal topology during differentiation necessarily requires redistribution of cohesin complexes across the genome [42,89]. Mechanistically, cohesin depletion represents functional removal from pluripotency-associated regions rather than elimination from the nucleus.

In parallel with these chromatin changes, differentiating ESCs undergo a profound shift in genome maintenance programs. As discussed above, pluripotent cells preferentially utilize HR during their extended S phase, whereas differentiated cells increasingly rely on non-homologous end joining for DNA repair. This transition is accompanied by reduced expression of HR-factors and altered cohesin localization, suggesting that diminished demand for sister chromatid-mediated DNA repair may further contribute to decreased cohesin retention on chromatin. Thus, differentiation simultaneously reduces the structural and functional requirements for high levels of cohesin-mediated chromosome organization.

Collectively, current evidence supports a model in which cohesin reduction during differentiation is driven by coordinated chromatin remodeling rather than by simple transcriptional downregulation of cohesin subunits. Chromatin compaction, attenuated enhancer activity, altered NIPBL-WAPL balance, and reduced HR redistribute cohesin from pluripotency regions to establish lineage-specific architecture. This dynamic regulation enables differentiating cells to replace the highly plastic chromosomal organization required for self-renewal with a more stable lineage-specific genome organization.

### 6.2. Kleisin Subunit-Specific Lineage Determination

Perhaps the most intriguing finding regarding cohesin’s role in ESC biology is the discovery that knockdown of different cohesin subunits directs differentiation toward distinct cell lineages [19]. Gene set enrichment analysis (GSEA) of RNA-seq data from SMC3-depleted ESCs revealed enrichment of gene sets associated with connective tissue development, neuron differentiation, and germ cell development, indicating that loss of the core cohesin subunit promotes multi-lineage differentiation [19]. More strikingly, the two α-kleisin subunits RAD21 and REC8 induce distinct differentiation trajectories when depleted. REC8-depleted ESCs show enrichment of genes involved in vasculature development and angiogenesis, with leading-edge genes including CCN1, a regulator of endothelial tip cell activity, CCN2/CTGF, which promotes vascular endothelial cell differentiation, and SERPINE1, a key regulator of angiogenesis [19]. In contrast, RAD21-depleted ESCs exhibit strong enrichment of genes associated with neurogenesis and gametogenesis [19]. Specifically, the siRAD21 condition shows upregulation of neural lineage markers, including genes such as SCN1B (involved in action potential generation in neurons) and NFIB (essential for neural stem cell differentiation), alongside germline markers such as MOV10L1 (a key component for maintaining genetic information in male germ cells) and PIWIL2 [19].

Validation experiments using quantitative PCR and Western blotting have confirmed these lineage-specific differentiation patterns. Neural markers NES and NEUROD1 are expressed at 2.46-fold and 1.37-fold higher levels, respectively, in differentiated siRAD21 cells compared to siREC8 cells, while germline markers STRA8 and DDX4 show 2.57-fold and 2.74-fold increases [19]. These data demonstrate that the α-kleisin subunit composition of cohesin influences not only whether ESCs differentiate but also the specific lineage to which they commit. This finding has significant implications for regenerative medicine, as it suggests that manipulation of specific cohesin subunits could provide a novel approach for directing ESC differentiation toward desired cell types.

### 6.3. Cohesinopathies—Developmental Disorders Linked to Cohesin Dysfunction

The importance of cohesin in human development is underscored by a group of multisystem developmental disorders, collectively termed cohesinopathies, that arise from germline mutations in cohesin subunits or their regulators [81,96]. The most prevalent cohesinopathy is Cornelia de Lange syndrome (CdLS), which is characterized by growth retardation, limb abnormalities, facial dysmorphism, and intellectual disability [97]. The majority of CdLS-associated mutations occur in NIPBL, the cohesin loading factor, though mutations in SMC1A, SMC3, RAD21, and HDAC8 have also been identified [97,98]. The pathogenesis of cohesinopathies is thought to result primarily from altered gene expression rather than defective sister chromatid cohesion [99]. NIPBL mutations reduce the dose of cohesin on chromatin, leading to global decreases in DNA loops and domains and misexpression of thousands of genes important for development [100]. Recent studies have shown that decreasing WAPL dosage can partially rescue the transcriptional defects caused by NIPBL haploinsufficiency, consistent with a model in which the balance between cohesin loading and release determines the level of cohesin on chromosomes and consequently the pattern of gene expression [101,102]. However, WAPL reduction does not fully rescue the developmental phenotypes, suggesting that additional cohesin functions beyond loop extrusion contribute to the disease pathology [102].

Individual cohesin subunits appear to have specialized roles in specific developmental contexts. STAG2 loss in oligodendrocytes alters enhancer–promoter communication and expression of myelin-promoting genes, potentially explaining the neurological phenotypes observed in cohesinopathy mouse models [103]. RAD21 loss in postmitotic neurons reduces long-range DNA interactions necessary for the expression of late response neuronal genes [104]. These findings are consistent with the observation that RAD21 depletion in ESCs promotes differentiation toward neural lineages [19], suggesting that cohesin plays a particularly critical role in neural development.

### 6.4. Cohesin Mutations in Cancer

Cohesin is among the most frequently mutated protein complexes across all cancer types [105]. Somatic mutations in cohesin subunits (SMC3, RAD21, STAG2, STAG1) and regulators have been identified in a wide range of malignancies, with STAG2 being particularly frequently mutated [105,106]. Since complete loss of cohesin is lethal, cancer-associated mutations are typically partial loss-of-function alterations that affect the dose of cohesin on chromatin or the efficiency of specific cohesin activities [107]. A point mutation in SMC1A (R586W), identified in cancer, was shown to reduce cohesin localization to enhancers and promoters but not CTCF sites, leading to a global loss of short-range DNA contacts [108].

The synthetic lethal relationship between STAG1 and STAG2 represents a promising therapeutic avenue. Because STAG1 and STAG2 are mutually exclusive cohesin subunits with partially overlapping functions, cells that have lost STAG2 become dependent on STAG1 for viability [82,109]. This dependency creates a vulnerability that could be exploited therapeutically in STAG2-mutant cancers. Additionally, the 3D genome organization of cancer cells can be hijacked to promote oncogenesis through structural rearrangements that alter enhancer–gene relationships [83]. CTCF binding sites are enriched for sequence variation in cancer genomes, and mutations at these sites can alter DNA loop propensity and consequently gene expression [110].

To provide readers with a concise overview of the clinical relevance of cohesin alterations, the major cohesin gene mutations, their associated cancer types, approximate mutation frequencies, and functional consequences are summarized in Table 2. Across tumor types, cohesin genes are recurrently affected by predominantly loss-of-function mutations, with STAG2 being the most frequently altered subunit; the spectrum ranges from high mutation frequencies in urothelial bladder carcinoma and Ewing sarcoma to recurrent involvement in myeloid malignancies, where cohesin mutations define a distinct molecular subset [84,85,110,111]. Ultimately, understanding how cohesin mutations reshape the 3D genome in cancer cells will be critical for developing targeted therapeutic strategies.

## 7. Discussion

The cohesin complex has evolved from its original characterization as a simple molecular glue for sister chromatids into one of the most versatile and multifunctional protein complexes in chromosome biology. In ESCs, cohesin performs an exceptionally diverse array of functions: it maintains sister chromatid cohesion essential for accurate chromosome segregation, facilitates DNA loop extrusion that organizes the 3D genome, supports the HR-mediated DNA repair pathway critical for genome integrity during rapid proliferation, and helps maintain the transcriptional programs underlying pluripotency and self-renewal.

The discovery that meiosis-specific cohesin components REC8 and STAG3 are functionally active in mitotic ESC chromosomes has opened new avenues of investigation. The co-existence of distinct cohesin populations, RAD21-containing mitotic cohesin and REC8-containing meiotic cohesion, in the same cell, each with unique chromosomal loading patterns and functional properties, adds an additional layer of complexity to chromosome organization in ESCs. The observation that the balance between these two cohesin types can be dynamically adjusted, with REC8-cohesin compensating for RAD21-cohesin loss, suggests a remarkable adaptability in the cohesin system that may be critical for ESC resilience. The interplay between cohesin and condensin in regulating chromosome topology represents another frontier. The shared genomic binding sites of these two SMC complexes and the consequence of cohesin loss for condensin distribution and chromosome compaction highlight the need for a more integrated understanding of how multiple SMC complexes coordinate to shape chromosome structure throughout the cell cycle. In ESCs, where both mitotic and meiotic cohesins coexist with condensin, this coordination is likely to be particularly intricate. Perhaps most exciting from a translational perspective is the finding that different cohesin subunits direct ESC differentiation toward distinct lineages. The ability of RAD21 depletion to promote neural and germline differentiation, and of REC8 depletion to favor vascular differentiation, suggests that targeted manipulation of cohesin composition could provide novel strategies for generating specific cell types for regenerative medicine applications. However, significant challenges remain, including the need to achieve more precise control over the degree and timing of cohesin perturbation and to validate these findings in human ESC and induced pluripotent stem cell systems.

Rather than accepting this as an established conclusion, these recent results need to be tested and validated more thoroughly. Two considerations complicate straightforward acceptance of a specific, non-redundant mitotic function for REC8/STAG3. First, the strongest genetic evidence for REC8 function in mice comes from in vivo *Rec8*-null models rather than cultured ESCs. These mice exhibit phenotypes strictly confined to meiosis, characterized by germ cell failure and infertility, a pattern consistent across all eukaryotic species examined to date [75]. That same study did report sub-Mendelian survival and failure to thrive in Rec8-null pups, a hint of an uncharacterized somatic requirement, but no dedicated ESC or inner-cell-mass proliferation, sister-chromatid-cohesion, or DNA-repair phenotype has been reported in that in vivo model. The severe mitotic phenotypes observed after acute REC8 or STAG3 knockdown in cultured ESCs conflict with the mild, meiosis-restricted phenotypes seen in genetic knockout models. Thus, these culture-based effects may reflect acute depletion stress or artifacts of the culture system rather than a physiological division of labor between RAD21 and REC8. Second, a simpler interpretation for the presence of REC8 and STAG3 in ESCs deserves consideration. Independent studies show that various germline-restricted genes, including cohesin subunits, are detectable in ESCs and iPSCs. This is largely driven by low-level transcription allowed by the open chromatin state of pluripotent cells, rather than a coordinated regulatory program [74]. Under this view, REC8 and STAG3 expression in ESCs represents a general phenomenon rather than a cell-type-specific cohesin pathway. Distinguishing between these two possibilities requires independent loss-of-function and structural evidence, which is currently lacking. These two interpretations are not mutually exclusive. Permissive, broad expression may have provided the baseline upon which a specific mitotic function for REC8/STAG3 evolved, though this hypothesis requires experimental validation.

Several key questions remain to be addressed in future studies. What are the precise molecular mechanisms by which different cohesin subunits influence lineage-specific gene expression programs? How do mitotic and meiotic cohesins divide their responsibilities in organizing the ESC genome, and what happens to this division during differentiation? What is the functional significance of the cohesin–condensin competition at shared genomic binding sites, and how does this competition influence cell fate decisions? Finally, can the insights gained from studying cohesin in ESCs be translated into therapeutic strategies for cohesinopathies and cohesin-mutant cancers? Addressing these questions will require the integration of advanced technologies, including single-cell multi-omics, live-cell super-resolution imaging, and precise genome engineering approaches. The answers will undoubtedly deepen our understanding of the fundamental principles governing chromosome organization and stem cell biology.

### Clinical Implications and Limitations

Evaluating the strength and generality of the current evidence is essential. The first limitation concerns the evidentiary base and its reproducibility. Essentially all current evidence for a mitotic role of REC8/STAG3 in pluripotent cells derives from mouse ESCs and, within that, from a small number of studies originating largely from a single laboratory. A mitotic function for meiotic cohesin has not yet been independently established in human ESCs or iPSCs. We therefore present the REC8/STAG3 mitotic-cohesin model as a strong but still narrowly replicated hypothesis whose acceptance must await independent, multi-laboratory confirmation.

The second limitation concerns why extrapolation from mice to humans cannot be assumed. First, cell-cycle architecture differs quantitatively between species; the extremely short G1 and very high S-phase fraction that demand elevated HR usage and a coupled cohesin pool are far more pronounced in mouse than in human ESCs. Second, the pluripotency state is a major variable; most mouse data derive from naïve ESCs, whereas conventional human ESCs/iPSCs are in the primed state, which systematically differs in global chromatin openness, X-chromosome dosage, and the regulation of germline/meiotic gene programs. A REC8/STAG3 program permissively expressed in naïve mouse ESCs may be silenced or non-functional in primed human cells due to species-specific epigenetic control. Third, technical confounds are non-trivial, as detecting low-abundance meiotic cohesin requires antibodies, degron alleles, and imaging pipelines that must each be independently validated in human cells to rule out cross-reactivity or over-expression.

These limitations condition the translational implications of these findings. From a translational perspective, the finding that different cohesin subunits bias ESC differentiation toward distinct lineages, where RAD21 depletion promotes neural and germline differentiation, and REC8 depletion favors vascular differentiation in mouse ESCs, is intriguing. However, these loss-of-function studies establish association rather than causation, as protein-level rescue experiments demonstrating sufficiency have not yet been performed. Thus, directing cohesin composition as a novel strategy for regenerative medicine remains a contingent possibility rather than a near-term application, and its validation awaits confirmation in human pluripotent systems alongside more precise control over the degree and timing of cohesin regulation.

An unresolved question is how different kleisin subunits produce distinct lineage biases despite sharing the same SMC core complex. RAD21- and REC8-containing cohesin complexes exhibit distinct chromosomal localization and dynamics in ESCs, indicating they regulate different genomic regions. Depletion of each kleisin may destabilize specific subsets of enhancer–promoter interactions, selectively activating lineage-specific transcriptional programs. Another possible explanation involves differences in chromatin dynamics. RAD21 cohesin exhibits rapid turnover and drives active loop extrusion and enhancer–promoter communication. Loss of RAD21 preferentially disrupts dynamic pluripotency networks, enabling activation of neural and germ-cell programs sensitive to 3D genome organization. In contrast, REC8 cohesin provides greater stability for DNA replication and chromosome maintenance. Selective REC8 depletion thus alters genomic regions involved in vascular development via changes in chromatin accessibility or replication-associated organization.

Differentiation involves extensive rewiring of enhancer landscapes rather than global transcriptional changes. Because cohesin stabilizes enhancer–promoter communication, kleisin-specific complexes may unequally maintain different enhancer repertoires. Under this model, lineage specification arises not from direct regulation of individual genes by REC8 or RAD21, but from selective disruption of higher-order interactions controlling regulatory networks. Genome-wide occupancy analyses combined with single-cell transcriptomics are required to determine whether RAD21- and REC8-cohesin complexes occupy distinct enhancer clusters governing differentiation pathways.

Answering these core questions, including the molecular basis of kleisin-specific lineage bias, cohesion–condensin competition, and the translation of ESC insights to cohesinopathies and cohesin-mutant cancers, will require the integration of advanced technologies. The future of the field depends on combining single-cell multi-omics, single-molecule force spectroscopy, high-speed atomic force microscopy, FRET reporters of the cohesin conformational cycle, cryo-electron microscopy, live-cell super-resolution imaging with single-particle tracking, polymer-physics modelling of loop extrusion, and precise genome engineering approaches. Distinguishing between proven facts and remaining questions gives the field a clear roadmap.

## Figures and Tables

**Table 1:** Subunit composition, dynamics, and functional roles of the cohesin complex.

Subunit	Class/Complex	Expression	Key Partners	Dynamics/Biophysics	Functional Role	Disease	References
RAD21	Mitotic α-kleisin	Ubiquitous	SMC1A/3, STAG1/2, WAPL, PDS5, NIPBL	Highly dynamic; WAPL-sensitive; minutes-scale residence	1. Closes ring 2. Loop extrusion 3. Separase-cleaved 4. ESC depletion → neural/germline bias	1. CdLS 2. Somatic mutations in AML, Ewing sarcoma	[18,80,81]
REC8	Meiotic α-kleisin; active in mitotic ESC	Meiosis; ESC	SMC3 (SMC1β in meiosis), STAG3	Stable, protease-protected; slow turnover; long residence	1. Separate SMC3–REC8 complex 2. Stable structural cohesion 3. Restrains condensing 4. ESC depletion → vascular bias	POI/infertility	[18,75,79,80]
STAG1	HAWK; mitotic	Ubiquitous	RAD21	Longer-residence at CTCF/telomeres	1. CTCF/telomere-proximal cohesion 2. Partially redundant with STAG2	Synthetic-lethal target in STAG2-mutant cancers	[82,83]
STAG2	HAWK; mitotic	Ubiquitous	RAD21	More dynamic; enhancer-enriched	1. Enhancer/CTCF engagement 2. Arm cohesion 3. Arrests extrusion via CTCF interface	Most frequently mutated cohesin gene	[83,84,85]
STAG3	HAWK; meiotic; active in ESC	Meiosis; ESC	REC8	Stabilizes & imports REC8; gates nuclear REC8	1. Required for meiotic cohesion/synapsis 2. ESC counterpart of STAG1/2	POI/infertility	[18,79,80]

**Table 2:** Major cohesin gene mutations in human cancer.

Subunit	Representative Cancer Type(s)	Approximate Mutation Frequency	Functional Consequence	References
STAG1	1. Rarely mutated 2. Therapeutic vulnerability	- Low frequency	1. Reduced chromatin-bound cohesin 2. Impaired loop formation and cohesion	[97,98]
STAG2	1. Urothelial carcinoma 2. Ewing sarcoma 3. AML/MDS 4. Glioblastoma	- Bladder: ~15–20% - Ewing sarcoma: ~15–22% - Myeloid: ~5–10%	1. Predominantly truncating loss-of-function 2. Impaired sister-chromatid cohesion and aneuploidy 3. Loss of CTCF/enhancer engagement with altered gene expression and differentiation block	[82,83,84,110,112]
RAD21	1. AML/MDS 2. Ewing sarcoma	~13% of cohesin-mutant myeloid cases	1. Reduced cohesin dosage on chromatin 2. Disrupted loop domains and impaired transcriptional/differentiation programs	[110,112]
SMC1A	1. AML/MDS 2. Colorectal carcinoma	~3% of cohesin-mutant myeloid cases	Missense variants (R586W) reduce cohesin at enhancers/promoters and cause global loss of short-range DNA contacts	[81,112]
SMC3	AML/MDS	~3% of cohesin-mutant myeloid cases	1. Reduced chromatin-bound cohesin 2. Impaired loop formation and cohesion	[110,112]
PDS5B/NIPBL	1. AML/MDS 2. Various carcinomas	~2% (PDS5B) of cohesin-mutant myeloid cases	Altered cohesin loading, maintenance, and residence dynamics, changing the level of chromatin-bound cohesin	[112]

## Data Availability

No new data were created or analyzed in this study. Data sharing is not applicable to this article.

## Abbreviations

ESCs, embryonic stem cells; mESCs, mouse ESCs; MEFs, mouse embryonic fibroblasts; TADs, topologically associating domains; HR, homologous recombination; NHEJ, non-homologous end joining; DSB, double-strand break; CdLS, Cornelia de Lange syndrome; POI, premature ovarian insufficiency; SMC, structural maintenance of chromosomes; STAG, stromal antigen; E–P, enhancer–promoter; 3D-SIM, 3D structured illumination microscopy; ChIP-seq, chromatin immunoprecipitation sequencing; FRET, Förster resonance energy transfer; AML, acute myeloid leukemia; MDS, myelodysplastic syndromes.
